# Prioritized Identification of Fearful Eyes during the Attentional Blink Is Not Automatic

**DOI:** 10.3390/brainsci13101392

**Published:** 2023-09-29

**Authors:** Shuaixia Li, Bin Hao, Wei Dang, Weiqi He, Wenbo Luo

**Affiliations:** 1 Research Center of Brain and Cognitive Neuroscience, Liaoning Normal University, Dalian 116029, China; lsx91@lnnu.edu.cn (S.L.); hewq@lnnu.edu.cn (W.H.); 2 Key Laboratory of Brain and Cognitive Neuroscience, Liaoning Province, Dalian 116029, China

**Keywords:** eye region, attentional blink, rapid serial visual presentation, attentional resources

## Abstract

The eye region conveys considerable information regarding an individual’s emotions, motivations, and intentions during interpersonal communication. Evidence suggests that the eye regions of an individual expressing emotions can capture attention more rapidly than the eye regions of an individual in a neutral affective state. However, how attentional resources affect the processing of emotions conveyed by the eye regions remains unclear. Accordingly, the present study employed a dual-target rapid serial visual presentation task: happy, neutral, or fearful eye regions were presented as the second target, with a temporal lag between two targets of 232 or 696 ms. Participants completed two tasks successively: Task 1 was to identify which species the upright eye region they had seen belonged to, and Task 2 was to identify what emotion was conveyed in the upright eye region. The behavioral results showed that the accuracy for fearful eye regions was lower than that for neutral eye regions under the condition of limited attentional resources; however, accuracy differences across the three types of eye regions did not reach significance under the condition of adequate attentional resources. These findings indicate that preferential processing of fearful expressions is not automatic but is modulated by available attentional resources.

## 1. Introduction

During the COVID-19 pandemic, face masks emerged as one of the most economically practical protective measures employed worldwide to mitigate viral transmission, becoming an essential item for daily travel. Notably, because wearing a mask can block the nose and mouth regions of the face, existing evidence suggests, on the one hand, that wearing face masks restrains the extraction and processing of facial information, leading to a decrease in the speed and accuracy of facial expression recognition [1,2,3]. On the other hand, wearing a face mask makes people pay more attention to information in the eye region, resulting in increased activity of the orbicularis oculi muscle and an expanded range of direct gaze perception [4,5]. Regarding the eye region, much popular literature refers to the eyes as “a window into the soul”. Empirical research in psychology has consistently revealed that during face perception tasks, the eye region tends to be prioritized for attention and receives the longest fixations within the facial structure [6,7]. Furthermore, the eye region represents one of the most informative sources for facial expression recognition, and people can accurately identify others’ current emotions and motivations based on isolated eye region cues. For instance, numerous studies have shown that basic facial expressions and a wide range of complex emotions can be recognized quickly and efficiently from images of isolated eye regions [8,9,10,11]. In particular, individuals from Eastern cultures exhibit a higher degree of reliance on the eye region to express and recognize emotional states from the face than do their Western counterparts [12,13]. Therefore, the eye region plays a crucial role in the perception of facial expressions. However, the perceptual and encoding processes underlying the perception of emotional cues in the eyes remain unclear.

It has long been known that people have evolved to prioritize perceptual analysis and elaborate encoding of visual stimuli with high-value signals while ignoring task-irrelevant stimuli because of the limited capacity of the attention system. This process is referred to as selective attention [14]. Facial expressions, which are highly prevalent expressive nonverbal social cues in daily life, have been shown to influence selective attention [15,16,17,18]. More specifically, both happy and threat-related fearful and angry expressions can rapidly capture attention and obtain prioritized processing compared to neutral expressions, as reflected by higher recognition accuracies and shorter reaction times [8,19,20,21]. Even when presented using isolated eye region cues, angry and fearful expressions can still be detected and recognized quickly and accurately, triggering pronounced threat detection advantage and increased neural activities [10,22,23,24]. Combining the classical spatial attention paradigm of dot-probe, Carlson and colleagues found that regardless of the conscious awareness of eye region stimuli, fearful eye regions modulate spatial attention by facilitating attentional orienting and delaying attentional disengagement [25,26], supporting the notion of high sensitivity of the human brain to emotional cues in the eye region [27,28].

In addition to selective attention in the spatial domain, selective attention in the temporal domain focuses on the attentional processing of sequentially presented stimulus streams. A classical task for investigating this issue is the dual-target rapid serial visual presentation (RSVP), which permits a detailed exploration of the impact of available attentional resources on the processing of predefined targets [29,30]. In this task, a widely acknowledged phenomenon is the attentional blink (AB), in which the recognition accuracy of the second target (T2) significantly decreases when it appears within 200–500 ms after the presentation of the first target (T1) [31,32]. When emotional words [33,34,35], scene pictures [36,37], or facial expressions [38,39,40,41] are used as T2 and neutral stimuli as T1, an increasing number of studies have revealed that positively and negatively valenced T2 stimuli presented within the AB period are associated with significantly higher accuracy than did neutral T2 stimuli. Considering the essential importance of the eye region in social interactions [42], we recently presented happy, neutral, or fearful eye regions as T2 and observed reduced AB effects induced by happy and fearful eye regions [23,24]. These findings strongly support the notion that the emotional significance of T2 stimuli facilitates prioritized access to the capacity-limited attentional system, allowing for fine-grained encoding and representation at the working memory stage [16,43].

Beyond the influence of the emotional valence of T2 stimuli, several studies have revealed that the perceptual relevance between two targets or between targets and distractors affects AB [30,44,45,46]. That is, the more similar the T2 perceptual attributes are to the preceding T1 or distractor, the stronger the AB effect. Based on multiple channels for configural and featural processing, the AB effect is assumed to occur when the processing channels or routes involved in the discrimination of T1 and T2 stimuli overlap. Importantly, in addition to the feature-based channel used in object discrimination, a configural-based channel is available for the discrimination of facial stimuli. Following this logic, non-face T1 and face T2 stimuli do not compete for a single capacity-limited resource pool, which might lead to attenuation or absence of the AB effect for face T2 presented during the AB period [47]. Our recent studies that focused on the relationship between temporal attention and the processing of eye region expressions repeatedly showed that happy and fearful eye regions could reduce the AB effect, regardless of whether the eye region of emotions was the first or second of the two targets; one of three neutral house images was displayed as the other target [23,48]. The differences in perceptual attributes and processing channels between both targets may partially account for the attenuated AB effect. Nevertheless, little is known about the influence of the emotional content of T2 on AB when the same processing channel is recruited.

The present study aimed to resolve the concerns raised above and further investigate whether emotional eye regions serving as the T2 still can reduce the AB effect when T1 and T2 belong to the same face processing channel. To this end, we carried out a dual-target RSVP task in which upright eye region images of humans, apes, and dogs with similar physical features were presented as the T1 stimuli and upright happy, neutral, or fearful eye region images of humans were presented as the T2 stimuli, and 12 images of inverted neutral eye regions were presented as distractors. The stimulus onset asynchrony (SOA) between T1 and T2 was set to either 232 or 696 ms so that T2 appeared at lag2 or lag6. This experimental protocol was similar to that used in our previous work [23,40], which could elucidate the effect of perceptual similarity between two targets on the AB to some degree. After the presentation of an RSVP stream, participants were asked to recognize the source of the T1 eye region and the emotional category of the T2 eye region successively. Increasing evidence supports the idea that T2 accuracy within the AB period is significantly lower than that outside the AB period [30,31,49]; the same holds true for emotionally valenced eye regions acting as T2 [23]. Consistent with these observations, we hypothesized that the accuracy of recognition of the emotions conveyed by eye regions in T2 in the lag6 condition would be higher than that in the lag2 condition. Furthermore, several studies have reported that an increase in the perceptual load of the T1 can reduce the detection rate of fearful T2 faces [50,51], which reflects that the privileged processing of fearful faces does not occur automatically but relies on available attentional resources. Since T1 and T2 in the present study were eye region stimuli, they would occupy the same processing channels and compete for limited central cognitive resources. Thus, reducing attentional resources would be expected to inhibit the prioritized processing of fearful eye regions in the lag2 condition and give rise to less accurate accuracy.

## 2. Materials and Methods

### 2.1. Participants

Twenty-eight (20 female) college students were recruited from Liaoning Normal University. Participants age was between 18 and 25 years (M = 20.46 years). This sample size was sufficient to test the effect of the emotional valence of eye regions on the AB at a medium effect size level (Cohen ƒ = 0.25, power = 0.95, α error = 0.05). All participants were right-handed, with normal vision or corrected vision, and had no history of brain or psychological disorders. They had not previously participated in similar experiments and received appropriate monetary compensation after the experiment.

### 2.2. Stimuli

From the Chinese Facial Affective Picture System [52], 15 neutral faces (8 female), 2 happy faces, and 2 fearful faces (half female) were selected as the original materials. Furthermore, high-definition images of 1 ape and 1 dog were selected from the internet. Adobe Photoshop 8.0 software was used to crop and grayscale the human or non-human images mentioned above, transforming them into elliptical images showing only the eye region visible, with a size of 252 pixels wide and 100 pixels high. Among these eye region pictures, 2 happy, 2 neutral, and 2 fearful upright human pictures were used as T2 stimuli, 12 neutral pictures (6 female) from human face models were used as distractors and presented inversely, and the remaining 3 upright pictures (1 ape, 1 dog, and 1 female) were used as the T1 stimuli. When participants viewed these images from a chair located 65 cm away from the display screen, the visual angle was 5.9° × 2.3°. The brightness, contrast, and other low-level physical features of all stimuli images were standardized.

In our pilot study, twenty-two participants (15 female, mean age = 21.68 years) from Liaoning Normal University were recruited to rate the valence and arousal of 6 eye region images (T2) using a 9-point scale. Rating results showed that three types of eye regions (happy, neutral, and fearful) differed significantly from one another in valence [*F*(2, 42) = 259.21, *p* < 0.001, *η_p_*^2^ = 0.92]. As shown in Table 1, the happy eye regions were assessed more positively than the neutral (*p* < 0.001) and fearful (*p* < 0.001) eye regions and the fearful eye regions were assessed more negatively than the neutral eye regions (*p* < 0.001). Regarding arousal, a significant main effect of the emotional category was observed [*F*(2, 42) = 76.60, *p* < 0.001, *η_p_*^2^ = 0.78]. Further pairwise comparisons revealed that the happy and fearful eye regions were rated as more arousing than the neutral eye regions (*p*s < 0.001), while the difference between the former two conditions was not significant (*p* = 0.17). 

### 2.3. Procedure

**Figure 1:**
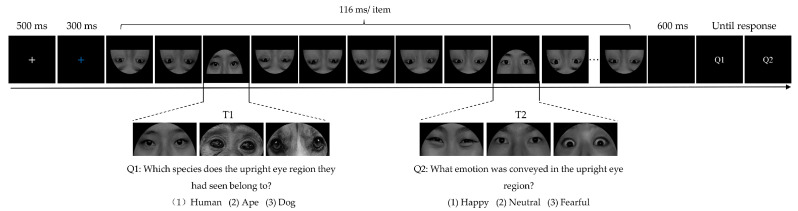
Illustration of a typical experimental trial sequence.

The experiment was conducted in a quiet cognitive-behavioral laboratory. Participants were seated in a comfortable chair positioned 65 cm away from the 19-inch computer monitor. The screen resolution was 1440 × 900 pixels with a 60 Hz refresh rate. The experimental procedure was programmed using E-Prime 2.0 software (Psychology Software Tools, Inc., Pittsburgh, PA, USA). As displayed in Figure 1, at the beginning of each trial, a white fixation cross was presented at the center of the screen for 500 ms, followed by a blue fixation cross for 300 ms, indicating the imminent appearance of the stimuli that needed attention. Next, an RSVP stream consisting of 12 inverted distractor images and 2 target images was presented at the same spatial location successively. Each image was presented for 116 ms. Three eye regions from different species (human, ape, and dog) were presented as T1, and happy, neutral, or fearful eye regions from human models were presented as T2. To avoid any preparation effect on T1, the stimulus appeared randomly and equiprobably at the 4th, 5th, or 6th position within the stimulus stream. T2 randomly and equiprobably appeared in the second or sixth position after T1. After a blank interval of 600 ms, two question screens appeared, and participants were required to report the specific source of T1 (Question 1: Which species does the upright eye region they had seen belong to? 1: Human; 2: Ape; 3: Dog) and the emotional category of T2 (Question 2: What emotion was conveyed in the upright eye region? 1: Happy; 2: Neutral; 3: Fearful) by pressing the corresponding digit on the keyboard without time constraint. The participants were informed that both tasks were equally important and that they had to report T2 as accurately as possible while prioritizing the correct identification of T1. No feedback was given. The inter-trial interval was 600 ms. 

Participants were instructed to practice 24 trials before the formal experiment to ensure that they clearly understood and were familiar with the experimental procedure. During the practice stage, the participants were given timely feedback on correct or incorrect responses. The formal experiment was divided into 4 blocks, resulting in 288 trials in total, and there were 48 trials for each condition. In each block of 72 trials, the number of conditions was equal, and the order was randomized. After completing a block, the participants were allowed to take a break for an appropriate amount of time before proceeding to the next block.

## 3. Results

### 3.1. Analysis of T1 Accuracy

The mean percentage of T1 accuracy across all conditions was 0.96 ± 0.01 (M ± SE). A two-way repeated measures analysis of variance (ANOVA) containing the within-subject factors of lag (2 levels: lag2, lag6) and emotional valence of the eye region (3 levels: happy, neutral, fearful) was run on the T1 accuracy. No significant main effects of lag [*F*(1, 27) = 0.63, *p* = 0.43, *η_p_*^2^ = 0.02] and emotional valence [*F*(2, 54) = 0.75, *p* = 0.47, *η_p_*^2^ = 0.03] were found, nor was there a significant interaction effect [*F*(2, 54) = 1.05, *p* = 0.36, *η_p_*^2^ = 0.04]. These results indicate that the accuracy of T1 was immune to the impact of T2 condition. 

### 3.2. Analysis of T2 Accuracy

**Figure 2:**
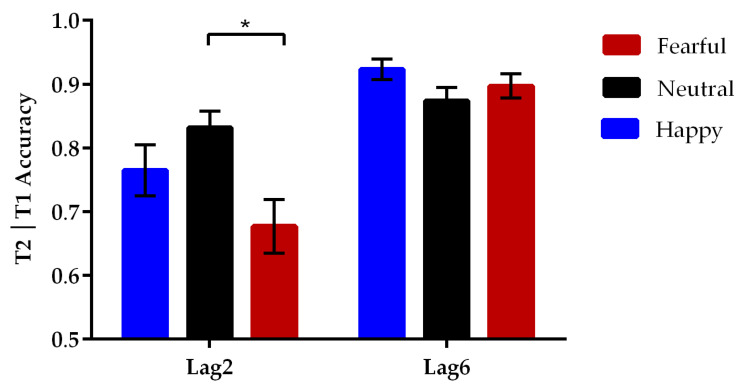
Mean percentage of correct identification of happy, neutral, and fearful T2 eye regions at each T1–T2 lag. Error bars represent standard errors of the means (* *p* < 0.05). The report of the fearful eye region was less accurate than that of the neutral eye region at the lag2 condition.

Since the source of T2 identification errors in incorrect T1 trials was unknown, the analysis of T2 accuracy focused only on trials wherein T1 was correctly reported [23,30]; see Table 2. A two-way repeated measures ANOVA on T2 accuracy was conducted with lag (2 levels: lag2, lag6) and emotional valence of the eye region (3 levels: happy, neutral, fearful) as within-subject factors. A significant main effect of lag was observed [*F*(1, 27) = 56.55, *p* < 0.001, *η_p_*^2^ = 0.68]. Further pairwise comparisons with Bonferroni correction revealed that the T2 eye regions in the lag6 condition (M ± SE, 0.90 ± 0.01) were identified more accurately than those in the lag2 condition (0.76 ± 0.02, *p* < 0.001). More importantly, there was a significant interaction between the lag and emotional valence [*F*(2, 54) = 11.76, *p* < 0.001, *η_p_*^2^ = 0.30], as seen in Figure 2. Post-hoc tests showed that the identification accuracy of neutral T2 (0.83 ± 0.03) was significantly higher than that of fearful T2 (0.68 ± 0.04, *p* = 0.012) during the condition of limited attentional resources (lag2), whereas the accuracy differences between the neutral and happy T2 trials (0.76 ± 0.04, *p* = 0.51) and between fearful and happy T2 trials (*p* = 0.23) were not significant. In the lag6 condition of adequate attentional resources, the accuracy of identifying happy (0.92 ± 0.02), neutral (0.87 ± 0.02), and fearful T2 (0.90 ± 0.02, *p*s > 0.14) did not differ significantly from one another. 

Furthermore, we analyzed the types of errors made when emotional T2 was incorrectly responded to explore the effect of response bias. Firstly, we calculated the percentage of trials on which T1 was correctly reported and T2 was incorrectly judged as either the neutral (Eneu) or emotional (Eemo) eye region. Then, a two-way repeated measures ANOVA was performed with error type (2 levels: Eemo, Eneu), lag (2 levels: lag2, lag6), and emotional valence of the eye region (2 levels: happy, fearful) as within-subject factors. As illustrated in Table 3, a significant main effect of lag was observed [*F*(1, 27) = 46.04, *p* < 0.001, *η_p_*^2^ = 0.63]. Further pairwise comparisons revealed that the rate of incorrect judgment for the T2 eye regions in the lag2 condition (M ± SE, 0.02 ± 0.003) was significantly higher compared to the lag6 condition (0.004 ± 0.001, *p* < 0.001). Additionally, a significant main effect of error type was observed [*F*(1, 27) = 26.22, *p* < 0.001, *η_p_*^2^ = 0.49]; incorrect judgments were more likely to be neutral (0.02 ± 0.003) rather than emotional (0.004 ± 0.001, *p* < 0.001). Moreover, there was a significant interaction between lag and error type [*F*(1, 27) = 24.32, *p* < 0.001, *η_p_*^2^ = 0.47]. Post-hoc tests showed that in the lag2 condition, the rate of T2 being incorrectly judged as neutral (0.033 ± 0.005) was significantly higher than that of emotional (0.006 ± 0.001, *p* < 0.001). Similarly, in the lag6 condition, the rate of T2 being incorrectly judged as neutral (0.007 ± 0.002) was also significantly higher than that of the emotional (0.001 ± 0.000, *p* = 0.001). 

## 4. Discussion

The human eye region has been found to transmit various emotional and social signals and play an important role in a large range of cognitive processes and adaptive behavior [42,53]. For instance, numerous studies indicated that not only fearful and angry expressions but also happy expressions projected in the eye region can attract attention rapidly and be processed efficiently [23,25,26,48]. However, it remains unclear whether the prioritized processing of fearful eye regions depends on available attentional resources. In this study, we selected neutral eye region images derived from three different species as T1 stimuli and emotionally valenced eye region images from humans as T2 stimuli. This was designed to enhance the perceptual similarity between two targets and maintain consistency in the processing channels. Furthermore, we manipulated the temporal interval between T1 and T2 as well as the emotionality of the T2 eye region to examine the influence of the affective valence of the eye region on AB. Here, we found a significant main effect of lag. Compared with the limited attentional resources condition of lag2, T2 accuracy in the adequate attentional resources condition of lag6 was significantly higher, indicating a close relationship between T2 accuracy and available attentional resources. Previous studies that used digits, letters, words, or facial expressions as T2 stimuli also systematically manipulated the temporal interval between T1 and T2 and consistently found a significant decrease in T2 recognition accuracy when T2 appeared 100–500 ms after the presentation of T1 [30,38,40,50,54]. In the present study, the lag2 condition represented a T1–T2 interval of 232 ms, while the lag6 condition represented a T1–T2 interval of 696 ms; the relatively higher recognition accuracy in the lag6 condition compared to the lag2 condition indicates that our manipulation of the T1–T2 temporal lag was appropriate and successfully induced an AB effect, providing new insight into the idea that attentional resources availability is a critical factor to influence the perception of visual stimuli.

Most importantly, there was a significant interaction between lag and emotional valence. Specifically, in the lag2 condition, the recognition accuracy for the neutral eye region was significantly higher than that for the fearful eye region, whereas no significant differences in recognition accuracy were observed between the happy and neutral eye regions or between the happy and fearful eye regions. In the lag6 condition, there were no significant differences in recognition accuracy among the happy, neutral, and fearful eye regions. In previous AB studies that used non-facial stimuli such as architectural images or letters as T1 and emotional facial expressions as T2, a widely accepted view was that emotional facial expressions, particularly threat-related expressions such as anger and fear, can rapidly capture attention and provide privileged access to awareness, leading to reduced AB effects [38,40]. Similarly, in two of our recent studies, in which neutral house pictures or neutral eye regions overlaid with five arrow pictures were used as T1 and emotionally valenced eye regions as T2, we also found that the recognition accuracies for happy and fearful eye regions in the lag2 condition were significantly higher than those in the neutral eye region [23,24]. These observations indicate that the emotional saliency of the T2 stimulus weakened the interference effect of T1. The results of the present study, which are contrary to previous findings, suggest that the prioritized processing of emotional stimuli may not be entirely automated and may be influenced by the availability of attentional resources and the congruency of processing channels for the two targets.

Previously, researchers systematically manipulated the perceptual load level of T1 stimuli and the emotional valence of T2 faces. They found that the detection advantage of fearful faces diminished or even disappeared under T1 high-load conditions [50,51], indicating that prioritized access of fearful faces to the limited-capacity cognitive system is not automatic and is sensitive to available attentional resources. Since the eye region is considered a diagnostic area for the recognition of fearful expressions [8,55], it has been shown that fearful eye regions can induce a threat detection advantage, similar to intact fearful faces [10,25]. In this case, the relatively lower accuracy for fearful relative to neutral eye regions in the condition of limited attentional resources in this study might suggest an inhibited processing advantage for fearful expressions, supporting the view that the processing of threatening stimuli requires a small number of attentional resources [50]. Furthermore, this result provides an important update to our understanding concerning how we perceive and encode the social signals derived from the eye region [53]. Simultaneously, the perceptual similarities between two targets and between targets and distractors can influence the magnitude of AB. In general, the AB effect increases with higher perceptual similarity between stimuli [30,56]. Unlike prior studies that used houses and intact facial expressions as T1 and T2 stimuli, respectively [38,40,56], both T1 and T2 employed eye-region stimuli in our study, resulting in higher perceptual similarity and shared processing channels. This setting may reduce the emotional significance of fearful eye regions and trigger a psychological refractory period [57], making it difficult for the perceptual representation of T2 to enter the central working memory consolidation stage successfully.

Furthermore, task switching between two targets as well as a response bias to report a neutral target, are potential factors that could influence the AB effect. Many studies have found that task switching involves time-consuming visual system representation reconstruction processes, during which the representation of T2 is delayed and attenuated to an unrepresentative level, leading to AB [58,59]. Moreover, the extent of system reconfiguration and cognitive resources consumed are closely linked to the relationship between the two targets. When two targets belong to the same category of stimuli, the cognitive system does not require reconfiguration. In contrast, the cognitive system needs to expend cognitive resources to reconfigure itself for efficient processing of the second target stimulus [60]. Thus, although eye region-eye region targets have a higher degree of perceptual similarity, the sources and task demands for these two targets are different. More cognitive resources of the same processing channel may be consumed during task switching, resulting in minimal cognitive resources being allocated to the T2 eye region, especially under conditions of limited attentional resources. With respect to the influence of response bias, Anderson, using a variety of dual-targets RSVP tasks, demonstrated that participants were more likely to guess the T2 as neutral words, especially when the T2 appeared within the AB period [33]. Similarly, our analysis of error types showed a pronounced response bias to report neutral expression. Considering our recent findings related to emotional eye region processing [23,24] and the absence of significant accuracy differences between neutral and happy conditions, it is possible that the neutral-oriented guessing strategy does not have the greatest impact on our results. Since the present study could not provide more information about the exact contributions of these factors to the AB, it may be better to see whether the present findings will be replicated in future investigations.

Two limitations of the present study need to be mentioned. First, considering the obvious individual differences in low-level physical features of the eye region, only two images for each kind of T2 stimuli were included. In this case, each picture was repeated 24 times in this study, which undoubtedly increased the familiarity of the stimuli and probably reduced the difficulty of the task. Second, it has been found that configural information in a face plays a crucial role in capturing attention and emotion recognition [61,62]. Previous studies often investigated the preferential processing advantage of fearful expressions with intact face images [17,40,50], but we only used isolated eye regions as experimental stimuli. In comparison with intact fearful faces, the perception of isolated, fearful eye regions is associated with reduced emotional saliency and inadequate configural encoding, which might hinder attentional capture and efficient recognition. Future studies with a great number of intact facial expression stimuli are necessary to replicate the current findings and further illustrate the relationship between emotional expression processing and temporal attention.

## 5. Conclusions

In conclusion, this study combined a dual-target RSVP task with emotionally valenced eye regions and found that the fearful eye region induced an increased AB effect compared with the neutral eye region under the condition of limited attentional resources, providing novel evidence for the prioritized processing of emotionally salient stimuli. Importantly, the preferential processing of fearful faces is not entirely automatic and depends on the availability of attentional resources and the congruency of processing channels.

## Figures and Tables

**Table 1:** Mean valence and arousal scores for happy, neutral, and fearful eye regions. Standard deviations are in parentheses.

Normative Ratings	Happy	Neutral	Fearful
Valence	8.07 (0.84)	4.00 (0.96)	1.70 (1.02)
Arousal	7.25 (1.36)	3.34 (1.28)	8.04 (1.67)

**Table 2:** Summary of the mean number of valid trials for different conditions. Standard deviations are in parentheses.

Condition	Both T1 and T2 Missed	Only T1 Reported	Only T2 Reported	Both T1 and T2 Reported
Lag2-Happy	0.68 (1.06)	9.43 (9.54)	1.18 (1.61)	36.71 (10.28)
Lag2-Neutral	0.50 (0.79)	6.07 (6.22)	1.50 (1.82)	39.93 (6.61)
Lag2-Fearful	0.93 (1.15)	13.21 (10.39)	1.32 (1.22)	32.50 (10.64)
Lag6-Happy	0.25 (0.70)	1.89 (2.79)	1.54 (2.03)	44.32 (4.04)
Lag6-Neutral	0.43 (0.88)	3.89 (4.13)	1.71 (1.88)	41.96 (5.35)
Lag6-Fearful	0.36 (0.91)	3.25 (3.12)	1.32 (1.98)	43.07 (4.89)

**Table 3:** Summary of the mean error rates for different conditions. Standard deviations are in parentheses.

Condition	Error Type	Error Rate
lag2-Happy	Neutral	0.028 (0.032)
	Fearful	0.005 (0.006)
lag2-Neutral	Happy	0.014 (0.014)
	Fearful	0.007 (0.010)
lag2-Fearful	Neutral	0.039 (0.035)
	Happy	0.007 (0.010)
lag6-Happy	Neutral	0.005 (0.010)
	Fearful	0.001 (0.003)
lag6-Neutral	Happy	0.009 (0.009)
	Fearful	0.004 (0.008)
lag6-Fearful	Neutral	0.010 (0.011)
	Happy	0.002 (0.003)

## Data Availability

The data are available from the corresponding author upon reasonable request.
